# An Infant Formula with Partially Hydrolyzed Whey and Intact Protein Demonstrates Adequate Growth and Safety: A 6-Month Randomized, Triple-Blind, Controlled Trial

**DOI:** 10.3390/nu18050770

**Published:** 2026-02-26

**Authors:** Qianqian Shen, Hua Jiang, Shuai Mao, Sha Luo, Yanjie Hao, Wenxin Liang, Tingchao He, Lotte Neergaard Jacobsen, Nan Sheng, Jing Yin, Xiaoying Feng, Xiaojiang Jia, Yvan Vandenplas, Yumei Zhang

**Affiliations:** 1 Department of Nutrition and Food Hygiene, School of Public Health, Peking University, Beijing 100191, China; qshen@stu.pku.edu.cn (Q.S.); 2011110167@bjmu.edu.cn (S.M.); wenxinliang@bjmu.edu.cn (W.L.); he_tingchao@bjmu.edu.cn (T.H.); 2 School of Nursing, Peking University, Beijing 100091, China; gail_ball@163.com; 3 Maternal and Child Health Hospital of Changsha County, Changsha 410100, China; 13549642685@163.com; 4 Department of Neonatology, Shahe People’s Hospital, Xingtai 054100, China; haoyanjie1127@163.com; 5 Strategic Business Unit Specialized Nutrition, Arla Foods Ingredients Group P/S, Viby J, 8260 Aarhus, Denmark; lojan@arlafoods.com (L.N.J.); jinyi@arlafoods.com (J.Y.); 6 Junlebao Dairy Joint Laboratory of Breast Milk Science and Life Health, Peking University, Beijing 100191, China; shengnan@jlbry.com (N.S.); fengxiaoying@jlbry.com (X.F.); jiaxiaojiang@jlbry.com (X.J.); 7 KidZ Health Castle, UZ Brussel, 1090 Brussels, Belgium

**Keywords:** partially hydrolyzed whey protein, infant formula, growth and development, growth trajectories, randomized controlled trial, breast feeding

## Abstract

Background/Objectives: Evidence suggests that partially hydrolyzed whey protein promotes appropriate infant growth; however, research on its long-term effects, especially in Asia, remains limited. This study set out to evaluate the effects of an infant formula containing partially hydrolyzed whey and intact protein on infant growth and development. Methods: This multicenter, triple-blind, randomized non-inferiority trial enrolled healthy full-term infants (≤14 days old). Participants were randomized (1:1) to receive pHF (*n* = 78) or SF (*n* = 70) until 6 months of age, with propensity score-matched exclusively breastfed (BF) infants (*n* = 70) serving as the reference. The primary outcome was daily weight gain. Linear mixed models assessed the association between feeding type and WHO z-scores over time. Results: Over 6 months, the adjusted mean (SE) daily weight gain (g) was 26.4 (1.27) g/day in BF, 26.0 (1.19) g/day in pHF, and 25.3 (1.27) g/day in the SF group. The adjusted mean difference between pHF and SF was 0.64 g/day (95%CI: −1.55, 2.83), confirming non-inferiority. Growth parameters were comparable between pHF and SF, with WHO z-scores remaining within ±1 SD of reference standards. Compared with pHF, SF was associated with a slower increase in length-for-age z score (LAZ). While there was no difference between the pHF and BF groups, WAZ increased significantly less in SF vs. BF [−0.34 (95%CI: −0.58, −0.10), *p* = 0.003]. Gastrointestinal disorders occurred more frequently in the SF group than in the BF group, with no significant difference between the pHF and BF groups. Conclusions: An infant formula containing partially hydrolyzed whey and intact protein supported adequate growth and was well tolerated during the first six months of life, with growth trajectories comparable to those of breastfed infants.

## 1. Introduction

Human milk (HM) is the best natural food for infants, providing essential nutrients and bioactive substances for growth and development [1]. The World Health Organization (WHO) and the United Nations Children’s Fund (UNICEF) recommend initiating breastfeeding within the first hour after birth and maintaining exclusive breastfeeding for the first six months of life [2]. However, global coverage remains suboptimal, with only about 44% of infants aged 0–6 months worldwide exclusively breastfed between 2015 and 2020 [3]. When exclusive breastfeeding is not feasible, bovine milk-based infant formula (IF) is commonly used as an alternative.

A key difference between HM and bovine milk lies in their protein composition, particularly the whey-to-casein ratio [4]. Mature HM typically contains about 60% whey and 40% casein, whereas bovine milk is dominated by casein (whey:casein, 20:80) [5]. To achieve a balanced protein composition, whey proteins are incorporated into IF [6]. Furthermore, previous studies have found that [7] HM contains inactive zymogens that are activated during suckling, generating proteases that partially hydrolyze intact HM proteins into peptides, thereby supporting digestion and gastrointestinal tolerance in infants. To emulate this physiological feature, partially hydrolyzed whey proteins have been introduced into IF. Such proteins possess an optimal amino acid profile, improved digestibility, and enhanced absorption compared with intact proteins [8].

A systematic review [9] and clinical trials [10,11,12,13] indicate that partially hydrolyzed whey protein formulas (pHF-w) are generally as safe as standard cow’s milk protein (CMP) formulas in terms of supporting normal growth and development. However, the existing evidence exhibits significant heterogeneity, involving varied study populations (preterm infants, allergy-prone infants, or older age at enrollment), short intervention durations, or formula with a low protein content and added functional components. There is a paucity of studies investigating the effects of pHF-w intervention on growth throughout early infancy, particularly during the first six months of life, in healthy full-term infants. Consequently, large-scale clinical trials are needed to confirm the benefits of routine pHF-w use in non-exclusively breastfed infants [14,15]. Moreover, as per European Commission regulations [16] applicable from 2021 onwards, which require clinical studies to demonstrate the safety and suitability of each specific hydrolysate-based infant formula, authorities do not accept the transfer of study results from one hydrolysate to another. Most previous studies have primarily utilized formulas based on 100% partially hydrolyzed whey protein [17,18], which may enhance digestibility but often influences taste and tolerance [19]. Furthermore, although China’s latest national food safety standard (GB 25596-2025) allows their use in infants with functional gastrointestinal discomfort, such formulations are generally regulated as formulas for special medical purposes rather than as standard infant formulas [20]. Therefore, formulas containing a moderate proportion of partially hydrolyzed whey protein may represent a more balanced approach, potentially improving digestibility and reducing the gastrointestinal burden compared to intact proteins, while better preserving palatability compared to 100% pHF-w, and the presence of intact protein fractions may contribute to the development of oral immune tolerance [21]. Importantly, strict adherence to the standard whey protein to casein ratio requirement (≥60:40) in infant formula is essential. Based on these considerations, we designed an infant formula containing ~40% partially hydrolyzed whey protein to balance palatability, nutrient profile, and tolerance.

This study aimed to assess the safety and growth outcomes of an infant formula containing approximately 40% partially hydrolyzed whey protein. We hypothesized that this formula, administered during the first six months of life, would be well tolerated and support adequate growth in healthy term infants, and demonstrate growth trajectories comparable to those observed in breastfed infants.

## 2. Materials and Methods

### 2.1. Study Design

The trial (Nutritional Intervention-Hydrolysate for comfort And Optimal growth, NIHAO) was a multicenter, triple-blind, parallel-group, randomized non-inferiority trial in 6 Chinese neonatal care units: five Women’s and Children’s Hospitals in Hunan province and one hospital in Hebei province. Infants were enrolled from 6 July 2023 to 25 March 2025, with intervention and follow-up assessments at 6 months (completed by 25 September 2025). The primary analysis time point was the 6-month follow-up visit. The trial protocol was approved by the Medical Ethics Research Board of Peking University (No. IRB00001052-20119), with governance approval granted at the site. The trial was completed with pre-registration at the China Clinical Trial Center (No. ChiCTR2100053122). The results are reported in accordance with the Consolidated Standards of Reporting Trials (CONSORT) reporting guideline. The protocol and prespecified statistical analysis plan were finalized before database lock and were consistent with the registered trial information.

### 2.2. Participants

Infant eligibility criteria included healthy, full-term, singleton infants born weighing between 2500 g and 4000 g and aged less than 14 days. For formula-fed infants, their mothers were required to be unable or voluntarily decided not to breastfeed. Meanwhile, breast-fed infants served as a reference group, and mothers were instructed to maintain exclusive breastfeeding for at least 4 months.

Infants with any of the following criteria were excluded: (1) preterm birth (<37 weeks’ gestation); (2) major congenital malformation either likely to interfere with the ability to ingest milk or affecting normal growth and development; (3) known cow’s milk protein allergy and other allergic diseases; or (4) mixed feeding (breastfeeding accounting for more than 40% of total feeding episodes in the formula-fed group, or formula feeding accounting for more than 20% of total feeding episodes in the breastfed group during the previous week). Written informed consent was obtained from all parents or guardians before randomization.

### 2.3. Randomization, Allocation Concealment, Blinding

After written consent was obtained, formula-fed infants were randomly allocated to either the intervention or control group in a 1:1 ratio through centralized stratified block randomization, with mode of delivery (vaginal or cesarean section) as the stratification factor and a block size of 4 in each stratum. The randomization schedule was generated by an independent statistician who was not otherwise involved in the trial. The block size was concealed from the research nurses to prevent predictability of allocation. Allocation concealment was ensured, as neither research nurses nor parents could predict the group assignment before enrollment. The intervention and control formulas were packaged identically, ensuring that study investigators and families remained blinded to treatment allocation throughout this study. Parents, research nurses, outcome assessors and data analysts were blind to the treatment group. Only the staff responsible for producing and managing the study formulas were aware of the group allocation. The data unblinding was performed after data analysis was completed. Emergency unblinding was permitted only if a serious adverse event was suspected to be related to the study formula.

### 2.4. Intervention

After enrollment, breastfed infants (BF group) continued to be breastfed exclusively for at least 4 months, and maternal diets were unmodified. Following randomization, formula-fed infants received one of the two formulas for 24 weeks. The intervention (pHF group) was a formula, composed of approximately 40% partially hydrolyzed whey protein and intact whey and casein with a protein content of 2.11 g/100 kcal. The partially hydrolyzed whey protein was Lacprodan^®^ IF-3070 (Arla Foods Ingredients Group P/S, Viby J., Denmark), with a degree of hydrolysis (DH) of 11% to 16%. The control (SF group) was intact whey protein and casein with a protein content of 2.17 g/100 kcal. In both formulas, whey protein accounted for ≥60% of total protein. All infants were fed on demand, and the use of non-study formulas was prohibited during the intervention period. Parents were required to bring back empty containers at each follow-up visit to assess adherence. Both formulas were produced and supplied by a Chinese dairy company (Junlebao Dairy Group Co., Ltd., Shijiazhuang, China), with the same appearance and color, identically packaged and stored. Formulas were manufactured according to the Food Safety National Standard of China (GB 10765-2021). Apart from protein hydrolysis, the two formulas were comparable in fat, carbohydrates, micronutrient composition, and energy density. The detailed nutrient composition of the two formulas is presented in Table 1.

### 2.5. Data Collection

Data were collected at baseline and 1, 2, 3, and 6 months of the intervention by uniformly trained investigators. Parents were required to bring their infant back to the hospital for each follow-up visit. To minimize loss-to-follow-up, a friendly reminder regarding the date of the next follow-up visit was given to each parent well ahead of time. Each visit involved a questionnaire and anthropometry indicators, including body weight, length, and head circumference. For all infants, the parents maintained a 3-day 24 h dietary record before each follow-up visit. And a food frequency questionnaire (FFQ) was conducted during the 3- and 6- month follow-up to collect dietary intake data. Anthropometric measurements were performed by well-trained research nurses at each site using standardized instruments provided centrally by the project team. All equipment was calibrated before each measurement session. Infant weight was measured using an electronic scale (SENSSUN, Zhongshan, China) while undressed, and recorded to the nearest 1 g. Length was measured in a supine position with a length board (SENSSUN, Zhongshan, China), accurate to 0.1 cm. Occipital–frontal head circumference was measured with a non-stretchable tape (FaSoLa, Yiwu, China) and rounded to the nearest 0.1 cm. Each measurement was taken three times, and the mean value was used for analysis. Z-scores for weight, length, and head circumference were derived based on WHO child growth standards [22]. A standardized written protocol and uniform printed training materials were provided to all sites. At each site, two trial coordinators were appointed to enroll and support parents in completing the questionnaires. Intra- and inter-observer reliability were periodically assessed throughout this study to ensure measurement consistency. Adverse events (AEs) and serious adverse events (SAEs) were collected through both active solicitation at scheduled visits and passive reporting by caregivers between visits.

### 2.6. Outcomes

All infants were managed according to the institutional protocols. The primary outcome was weight gain (g/day) from baseline until 6 months of intervention, calculated as the difference in infant weight between the baseline and the 6-month follow-up visits, divided by the number of days between these visits. We also calculated the weight gain from baseline to 3-month follow-up. Secondary outcomes included weight (kg), length (cm), and head circumference (cm) at baseline and at each follow-up visit, alongside their respective Z-scores, weight-for-age (WAZ), length-for-age (LAZ), weight-for-length (WLZ), and head circumference-for-age (HCZ), according to WHO Child Growth Standard z-scores [22]. Meanwhile, at the individual level, infant growth velocity from birth to 3 and 6 months of intervention was categorized as slow growth (z score difference < −0.67), within reference range (z score difference −0.67 to 0.67), or rapid (z score difference > 0.67) [23,24,25]. Tertiary outcomes included formula intake (assessed via a 3-day 24 h dietary recall for 3 consecutive days before each follow-up) and safety parameters.

### 2.7. Sample Size and Statistical Analysis

The sample size was estimated based on a non-inferiority design with a one-sided significance level of α = 0.025 and a type II error of β = 0.20 (power = 80%). According to a previous study [11,26], the non-inferiority margin (Δ) was set at −3 g/day, with an assumed standard deviation (σ) of 6.1 g/day. The expected mean difference (δ) in weight gain between the intervention and control groups after 3 months was 0.45 g/day. Under these assumptions, the required sample size was calculated to be 51 participants per group. Considering a potential dropout rate of 15%, the final target sample size was determined to be at least 59 participants per group.

Baseline characteristics were summarized using descriptive statistics. Continuous variables were tested for normality using the Shapiro–Wilk test and are presented as mean ± SD or median (IQR), depending on their distribution. Categorical variables are reported as percentages. The one-way ANOVA and the Kruskal–Wallis test, followed by Dunn’s post hoc test, were used to analyze continuous variables with normal distributions and skewed distributions, respectively. Categorical variables were compared using the χ2/Fisher exact test, as appropriate.

Propensity score matching (PSM) was performed to balance baseline characteristics between the BF group and the two formula-fed groups. Using the three groups as the grouping variable, PSM was performed for the BF group while retaining all participants in the two formula-fed groups. Considering the relatively high loss to follow-up in the BF group before the 1-month visit, infants who completed it were prioritized during PSM, while ensuring adequate covariate balance (maximum standardized mean difference [SMD] ≤ 0.2). Analyses were primarily conducted in the matched cohort.

The primary endpoint (weight gain during the six-month intervention in g/day) was analyzed using an analysis of covariance (ANCOVA) model, with the feeding groups as a fixed factor and adjustments for multiple covariates, including region, infant age at enrollment, sex, birth weight, birth head circumference, mode of delivery, gestational age at birth, maternal pre-pregnancy BMI, education level, and household income. Estimated least squares means (LS means) with standard errors (SE) are reported for each group, along with the adjusted mean difference and its 95% confidence interval (CI). Non-inferiority was considered established if the lower bound of the two-sided 95% CI for the difference in weight gain between the two groups (intervention minus control) was greater than −3 g/day.

In further analyses, linear mixed-effects models (LMMs) were used to evaluate longitudinal changes in WHO z-scores across different feeding groups. Crude models were first constructed, which included the different feeding groups, follow-up time, an interaction term of groups × follow-up time, and the random intercepts. The adjusted models were further controlled for the covariates mentioned above. Results are presented as β coefficients with 95% CIs. Potential confounders were identified from the literature or through differences in baseline characteristics among the three study groups. A sensitivity analysis was also performed by including complementary food intake as an additional covariate and by repeating the analyses in the original unmatched BF group.

Intent-to-treat analysis (ITT) was used for the main analysis, and no data was imputed for primary analysis. Data cleaning and statistical analysis were performed using R version 4.3.2. A 2-sided level of statistical significance was set at *p* ≤ 0.05.

## 3. Results

### 3.1. Baseline Characteristics

**Figure 1:**
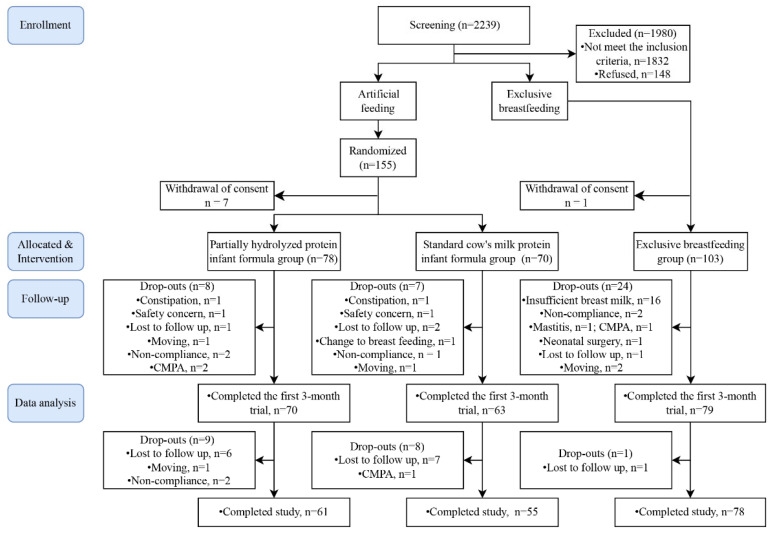
Study flow chart. CMPA: Cow’s milk protein allergy.

A total of 2239 screened infants across six centers were assessed for eligibility (Figure 1). Of the 259 eligible infants identified, 155 (60%) infants whose mothers were unable or voluntarily gave up breastfeeding were randomized. Seven participants withdrew informed consent after randomization; thus, 78 were assigned to the pHF group and 70 to the SF group. Additionally, 104 infants whose mothers intended to breastfeed exclusively were included in the BF group as a reference, and 1 participant withdrew informed consent after enrollment, leaving 103 infants in the final breastfeeding group. We assessed 212 infants (84.5%; 70 pHF, 63 SF and 79 BF) at 3 months and 194 infants (77.3%; 61 pHF, 55 SF and 78 BF) at 6 months. There was no statistically significant difference in the sample size recruited by each center among the three groups.

The maternal and neonatal baseline clinical characteristics were comparable between the two formula groups (Table 2). Overall, infants were enrolled at an average age of 8.10 days (SD, 6.46). The mean gestational age was 38.92 weeks (SD, 1.09), and the mean birth weight was 3232.24 g (SD, 399.44), with 53.21% vaginal deliveries, 56.88% males, and 34.40% primiparas. Statistically significant differences between the BF group and the two formula groups in age at enrollment, birth weight, head circumference, gestational age, mode of delivery, maternal education level and household income were observed. To address these imbalances, we performed PSM using the infant age at baseline, birth weight, gestational age and delivery mode as matching variables, and we successfully matched 70 infants in the BF group. After matching, no significant differences in infant baseline characteristics were detected among the three groups. All subsequent analyses were conducted using the propensity score-matched dataset.

### 3.2. Primary Outcome

**Figure 2:**
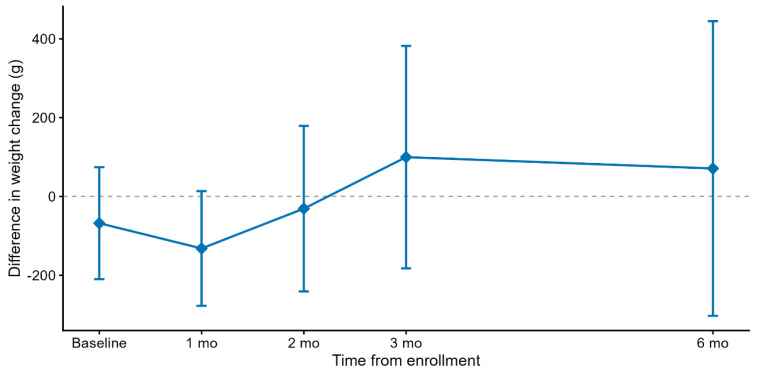
Adjusted difference in weight change between pHF and SF groups over time. Points plotted show the difference between the weight change in pHF and the weight change in SF, measured as the difference between weight at day of interest and weight at enrollment. Adjusted for region, infant’s gender, and age at enrollment. Error bars show the 95% CIs. Baseline is the enrollment date. pHF: Partially hydrolyzed whey protein infant formula; SF: Standard cow’s milk protein infant formula.

The adjusted mean (SE) daily weight gain over the six-month intervention was 26.0 (1.19) g/day in the pHF group and 25.3 (1.27) g/day in the SF group (Table 3). The between-group mean difference in weight gain was 0.64 (−1.55, 2.83) g/day, with the lower bound of the 95% CI above the predefined non-inferiority margin of −3 g/day. These findings demonstrate the non-inferiority of the intervention formula, indicating a comparable weight gain between groups. Similar results were observed during the three-month intervention period, with a mean difference of 1.05 (−2.16, 4.27) g/day. The mean (SE) weight gain from baseline to 3- and 6-month intervention was 39.9 (1.97) and 26.4 (1.27) g/d in the BF group, respectively. Given that there were no protocol deviations or any crossover between the groups, the per-protocol analysis was the same as the intention-to-treat analysis. The results of the sensitivity analyses before PSM were robust (Table S1). Furthermore, the difference in weight gain between the pHF and the SF groups showed that the two groups exhibited similar patterns of weight change across all follow-up time points (Figure 2).

A linear mixed-effects model was used to examine the changes in infant WHO z-scores over time (Table 4). Table 4 presents the associations of different feeding groups with WHO z-scores, respectively. Our findings suggest that the WAZ, LAZ, WLZ, and HCZ of all three groups of infants showed a linear increasing trend over time during the intervention period (*P_Time_* < 0.001 for all) regardless of whether the covariates are adjusted. Infants in BF group had significantly higher WAZ at baseline than those in pHF group in both crude (β = 0.33 [95% CI, 0.04 to 0.63]) and adjusted (β = 0.26 [95% CI, 0.01 to 0.50]) models. In addition, compared with pHF group, the SF group was associated with a slower WHO z score increase in LAZ (β = −0.18 [95% CI, −0.29 to −0.07] in the crude model; β = −0.17 [95% CI, −0.28 to −0.06] in the adjusted model). We also found that different feeding groups were not associated with WLZ or HCZ at baseline or with their changes over time in any model. Subsequently, interaction terms between group, time, and sex (or delivery mode) were added to the mixed-effects model. After adjustment for covariates, no significant three-way interaction was observed, suggesting that the effects of group and time on WHO z scores were similar across sex (or delivery mode) strata.

### 3.3. Secondary Outcome

Table 5 shows the overall adjusted pairwise mean differences (95% CI) in WHO growth standard z-scores among the three feeding groups after 6 months of intervention. After adjustment for relevant covariates, region, enrolled age, sex, birth weight, delivery method, delivery gestation age, maternal education level, and family financial status, infants in the SF group had a significantly lower WAZ (mean difference vs. BF = 0.34; 95% CI, 0.10 to 0.58) and HCZ (mean difference vs. BF = 0.49; 95% CI, 0.09 to 0.89) at the post-intervention time point. In contrast, after adjusting for covariates, no significant differences in WAZ, LAZ, WLZ, or HCZ were observed between the pHF and BF groups or between the pHF and SF groups (all *p* > 0.05), indicating that the post-intervention growth status of infants in the pHF group was comparable to that of both BF and SF groups. Notably, the pHF group consistently showed z-score values that were closer to those of the BF group across all four WHO indicators.

**Figure 3:**
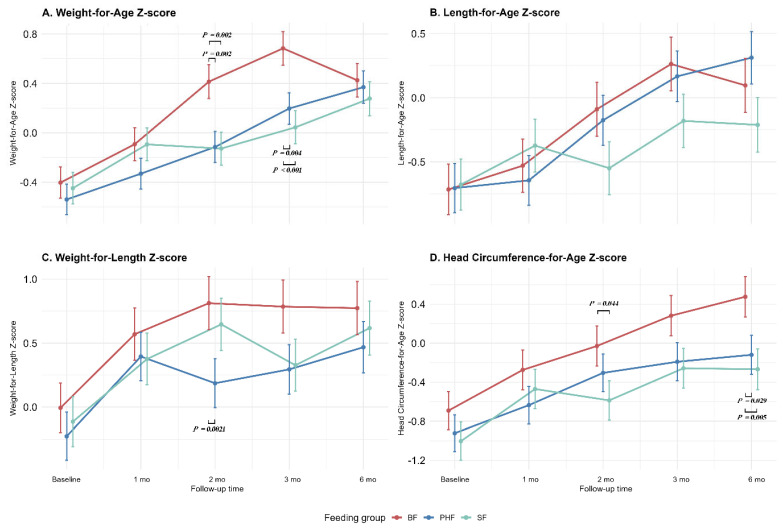
Adjusted estimated marginal means changes in WHO growth standard z-scores: weight-for-age (**A**), length-for-age (**B**), weight-for-length (**C**), head circumference-for-age (**D**) over time by different feeding groups. Values represent adjusted estimated marginal means derived from linear mixed-effects models with feeding group and time as fixed effects and infant as a random effect. Models were adjusted for region, enrolled age, gender, birth weight, delivery method, gestational age at delivery, maternal education level, and family economic status. Error bars indicate 95% confidence intervals. *p* values shown above the curves denote Tukey-adjusted pairwise comparisons between feeding groups at each follow-up visit. WHO, World Health Organization; pHF: partially hydrolyzed whey protein infant formula; SF: standard cow’s milk protein infant formula. BF: breastfeeding.

Furthermore, we conducted pairwise comparisons of WHO z-scores at each follow-up time point (Figure 3). Overall, BF infants consistently demonstrated more favorable growth profiles than SF infants, whereas pHF infants generally exhibited intermediate values and closely approximated the BF group across time. The unadjusted trajectories of WHO z-scores from baseline to 6 months are provided in Figure S1.

**Figure 4:**
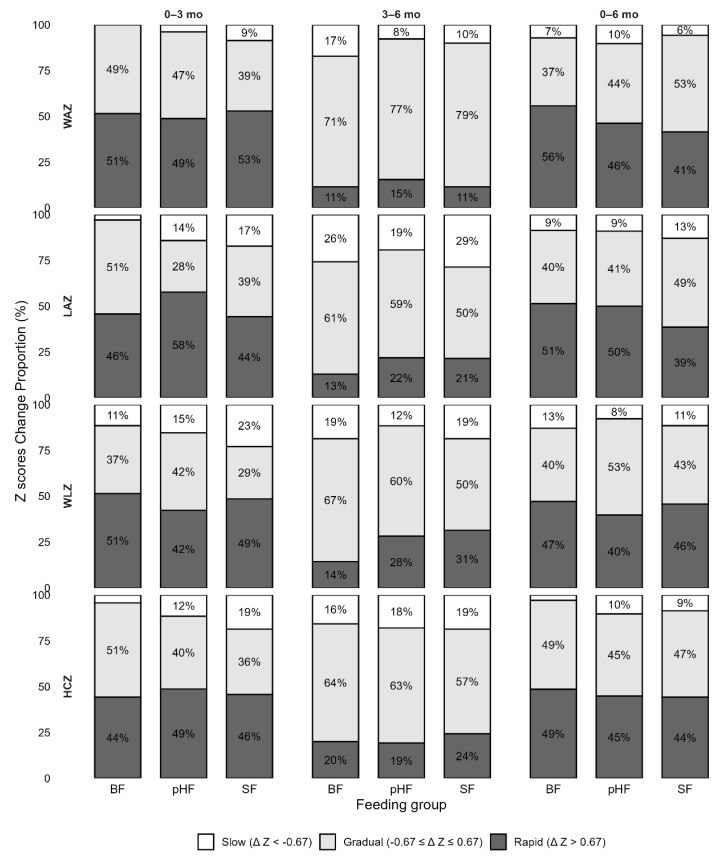
Rate of growth based on changes in WHO z-scores from baseline to 3 months, baseline to 6 months, and 3 to 6 months. WHO, World Health Organization; WAZ, weight-for-age z score; LAZ, length-for-age z score; WLZ, weight-for-length z score; HCZ, head circumference-for-age z score. pHF: partially hydrolyzed whey protein infant formula; SF: standard cow’s milk protein infant formula; BF: breastfeeding. The proportion <5% is not shown in the figure.

Growth velocity was further assessed by calculating the change in WHO z-scores between paired follow-up visits (from baseline to 3 months, 6 months, and 3 months to 6 months, respectively). For each of the growth parameters, we compared z scores at any of the two follow-up time points to measure an absolute change between the paired measurements. We used three baseline growth categories: reference range (z score difference, −0.67 to 0.67), rapid (z score difference, >0.67), and slow growth (z score difference, <−0.67). The proportions of growth velocity categories for each group in different time interval are shown in Figure 4. For LAZ, the change from baseline to 3 months differed significantly among feeding groups. As shown in the figure, gradual growth was observed in 51% of BF infants, compared with 28% in the pHF group and 39% in the SF group. BF infants demonstrated a lower proportion of slow growth compared with pHF (3% vs. 14%) and SF (3% vs. 17%). The differences in HCZ growth velocity among the three groups from baseline to 3 months were marginally significant (*p* = 0.071). No difference was detected between the other two groups or at other time points. 

During the intervention period, the average daily formula intake and energy intake were similar in both formula groups (*p* > 0.05) (Table 6). At the 3-month follow-up, infants in the pHF and SF groups consumed a mean (SD) of 881 (206) mL/day and 928 (224) mL/day of formula, respectively (*p* = 0.165). By 6 months, the corresponding mean (SD) daily intake was 946 (193) mL/day in pHF group and 933 (229) mL/day in the SF group (*p* = 0.743). We subsequently analyzed the FFQ collected at the 3- and 6-month follow-ups to compare the introduction of complementary foods among the three groups. Complementary foods were rarely introduced at 3 months. By the 6-month follow-up, 46.1%, 46.4%, and 38.6% of infants in pHF, SF and BF groups, respectively, had been introduced complementary foods. However, these differences were not statistically significant (*p* = 0.480). Therefore, complementary feeding was not included in the primary model, but was adjusted for in sensitivity analyses to confirm the robustness of the findings.

### 3.4. Adverse Events

**Figure 5:**
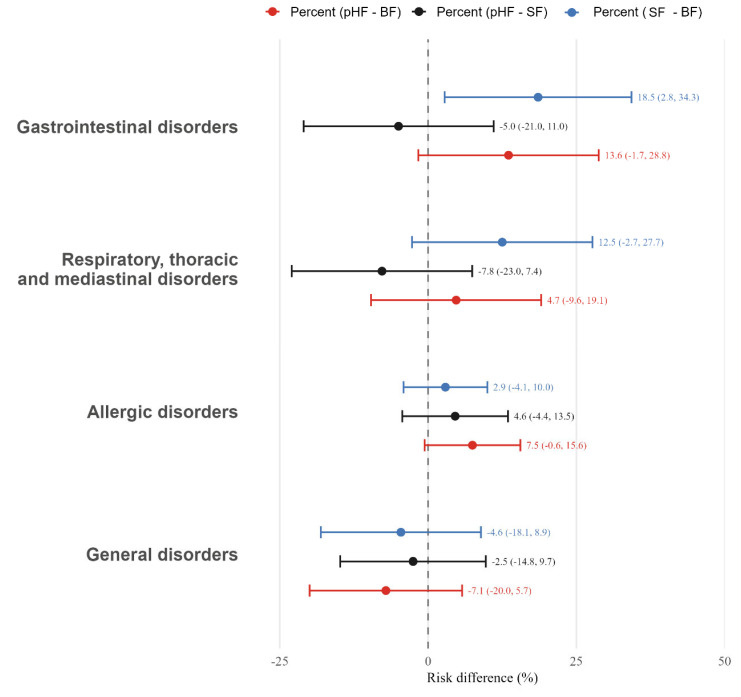
Forest plot with the estimated means as well as the mean difference (95%) occurrence of adverse events for each system organ class per/between intervention groups. pHF: partially hydrolyzed whey protein infant formula; SF: standard cow’s milk protein infant formula; BF: breastfeeding.

Adverse events in this study included gastrointestinal disorders (vomiting, regurgitation, food refusal, hematochezia, etc.), respiratory, thoracic and mediastinal diseases (cold, cough, pneumonia, bronchitis, etc.), allergic disorders (atopic dermatitis, allergic rhinitis, allergic asthma, etc.) and general disorders (fever and other). There were no statistically significant differences in the overall incidence of AEs throughout the intervention period, categorized by system organ class, between pHF and BF groups or between pHF and SF groups (Figure 5). None of the AEs were considered related to study formula consumption. Overall, 328 AEs occurred in the study population, where 130 of them occurred in the pHF group, 123 occurred in the SF group, and 75 occurred in the BF group. The most commonly observed AEs were gastrointestinal disorders and respiratory diseases. It is noteworthy that the incidence of gastrointestinal diseases was significantly higher in the SF group (46.9%) than in the BF group (28.3%), with a higher average number of gastrointestinal events observed in SF group (0.81 events) than in BF group (0.39 events) (Table 7). The incidence of respiratory diseases ranged from 25.0% (BF) to 37.5% (SF). No serious adverse events occurred.

## 4. Discussion

This multicenter, randomized study, evaluating the safety and tolerability of an infant formula containing partially hydrolyzed whey and intact protein in healthy full-term infants, demonstrated non-inferiority to a standard cow’s milk protein formula with respect to weight gain during the first six months of life. Furthermore, infants fed the SF showed a slower gain in LAZ over time compared with those receiving the pHF. In contrast, no significant differences in growth trajectories were observed between infants fed the pHF and those who were breastfed, suggesting that growth patterns associated with this partially hydrolyzed whey protein formula may be more comparable to those observed in breastfed infants.

To the best of our knowledge, this study represents the first randomized, triple-blind, controlled trial conducted in China to evaluate the effects of a 6-month intervention with partially hydrolyzed whey protein (approximately 40% of the total protein) on infant growth and development. Our findings are consistent with previous studies reporting no significant differences in daily weight gain between infants fed pHF and those fed SF or breast milk during early infancy, typically within the first 7 weeks to 3 months of life [13,27,28,29,30]. Notably, these studies involved diverse populations, including preterm infants, infants at high risk of allergy or healthy full-term infants. Wu et al. [27] reported comparable growth among healthy term infants fed a pHF-w, intact whey formula, or breast milk during the first three months of life; however, this was an open-label study and the test formula contained 63% whey protein, all of which was partially hydrolyzed, and 37% intact casein. Likewise, an RCT conducted in Greece [11] observed no significant differences in growth between infants consuming a pHF-w and those receiving a standard formula over 3 months, but did not include a breastfed reference group. By extending the intervention duration to six months, the present study provides more robust evidence that partially hydrolyzed whey protein supports adequate and sustained growth throughout early infancy, a conclusion further reinforced by sensitivity analyses using propensity score matching to account for baseline differences in infant characteristics. Notably, most previous pHF-w formulas [11,17,18] were composed exclusively of whey protein and lacked casein, a major protein in HM. While the European Food Safety Authority (EFSA) recognizes pHF-w as a nutritionally adequate protein source for infants [31], the inclusion of intact protein, as in the formula evaluated in our study, may more closely approximate the protein profile of human milk and support growth trajectories that better resemble those observed in breastfed infants.

In addition to daily weight gain, WHO child growth standard z-scores offer insights into longitudinal growth patterns. As expected, age-appropriate growth, with WAZ, LAZ, WLZ, and HCZ values within ±1 SD of WHO standards, was found in our three study groups [32]. Although baseline z-scores were comparable, growth trajectories diverged thereafter, with infants fed the SF showing slower increases in LAZ compared with those receiving the pHF, while growth trajectories in the pHF group were more closely aligned with those observed in breastfed infants. After 6 months of intervention, the only significant differences were higher WAZ and HCZ in the BF group relative to the SF group, a finding consistent with data from a Chinese national survey indicating that exclusively breastfed infants aged 1–6 months tend to have slightly higher body weight than partially breastfed or formula-fed infants [33]. Conversely, several studies suggested that SF may be associated with greater weight gain compared with breastfeeding. Wen et al. [34] reported that weight trajectories among infants fed pHF closely tracked those of breastfed infants, whereas infants consuming SF exhibited consistently higher mean body weights between 3 and 12 months of age. Julie et al. [35], in an analysis of z-score trajectories among infants aged 2.5 to 7.5 months, observed accelerated weight gain in SF-fed infants, while infants fed hydrolyzed formula demonstrated more normative growth patterns. Comparable findings have also been reported in infants at high risk of allergy [10]. Proposed explanations include higher protein intake in formula-fed infants and consequent effects on insulin-like growth factor-1-mediated growth [36,37], although findings have not been consistent across studies [38]. In our study, the protein content and intake were comparable between both formula groups, suggesting that the observed differences in growth trajectories may be more closely related to differences in protein digestion and absorption kinetics [39]. Partially hydrolyzed whey proteins, characterized by smaller peptide sizes, may facilitate faster gastric emptying and alter postprandial amino acid absorption profiles compared with intact cow’s milk proteins [40,41]. Given the established association between rapid z-scores trajectories and later obesity risk [42,43], the absence of excessive weight gain across feeding groups in our study may have potential clinical implications. In addition to protein characteristics, carbohydrate composition, particularly the presence of lactose versus alternative carbohydrate sources such as corn syrup solids, can influence infant postprandial metabolism and may be associated with divergent growth and adiposity outcomes [44,45]. However, in the present study, according to the Food Safety National Standard of China, both formula groups were predominantly lactose-based, thereby minimizing potential confounding due to the carbohydrate source.

Further insight was provided by an analysis of growth velocity categories, which indicated that differences between feeding groups were most pronounced during the early postnatal period, particularly for linear growth. During the first three months of life, breastfed infants exhibited a significantly higher proportion of gradual LAZ changes than pHF-fed infants, whereas infants in the SF group showed a higher proportion of slow LAZ increases, with marginal statistical significance. A systematic review shows that the HM protein concentration was positively associated with infant length, but no associations were reported for infant weight [5]. Positive associations have been reported between protein intake from all sources (HM, formula, and complementary foods) and infant growth in the first two years of life [46]. In our study, the proportion of rapid growth was numerically higher in the pHF group, although the difference did not reach statistical significance. Combined with the findings from the LMM analysis, LAZ trajectories in the pHF group appeared to increase at a faster rate than those in the SF group and were more closely aligned with the pattern observed in the BF group. One possible explanation may relate to differences in protein digestibility and absorption efficiency, with partially hydrolyzed protein potentially facilitating more efficient gastrointestinal processing. These findings are consistent with those of a recent combined analysis reporting greater monthly head circumference growth and length gains in infants fed pHF compared with those receiving SF [29], as well as with studies in infants at high risk of allergy, showing enhanced linear growth among those fed pHF [10]. However, the biological mechanisms underlying the effects of partially hydrolyzed protein on linear growth in early infancy remain insufficiently understood and warrant further investigation.

Throughout the study period, no statistically significant differences were detected between the two formula groups in either formula volume consumed or total energy intake, indicating that the observed differences in growth outcomes were unlikely to be driven by disparities in intake. Moreover, adjustment for the introduction of complementary foods did not materially alter the results, further supporting the robustness of the findings.

No significant differences were observed between the pHF group and either the BF or SF groups for the occurrence of adverse events, including gastrointestinal disorders, respiratory, thoracic and mediastinal disorders, allergic disorders, and general disorders. Nevertheless, a higher average number and rate of gastrointestinal AEs were observed in the SF group compared with the BF group, suggesting potential differences in gastrointestinal tolerance across feeding patterns. These findings are consistent with a possible beneficial effect of pHF on gastrointestinal comfort in early infancy. Previous evidence suggests that, relative to intact milk protein, partially hydrolyzed protein may demonstrate enhanced initial digestibility, reduced gastric curd formation, and accelerated gastric emptying, potentially conferring digestive advantages [9,47,48,49], and may contribute to more stable feeding patterns and nutrient utilization during early infancy, an observation that warrants further investigation.

This multisite study represents the first randomized clinical trial conducted in China to date to assess the effects of a partially hydrolyzed protein intervention on infant growth and development over six months, with high participant adherence and a low dropout rate, thereby enhancing the reliability of the findings. Clinicians, families, and outcome assessors were successfully blinded to group allocation, reducing the chance of bias arising in the clinical management or data collection. Additionally, we assessed several potential confounders to assess the robustness of our study findings. Growth velocity was assessed using both trajectory-based linear mixed-effects models and categorical changes in WHO z-scores, which capture distinct but complementary aspects of infant growth. The mixed-effects models evaluate differences in average growth trajectories across feeding groups, whereas the ΔZ-based classification identifies infants experiencing rapid or slow growth, a method widely used in prior studies and associated with later obesity risk [25]. Furthermore, we applied PSM to the non-randomly enrolled BF group to balance baseline characteristics across groups and minimize potential selection bias.

This study has several key limitations. First, this study was conducted in healthy, full-term Chinese infants with follow-up limited to the first 6 months of life; the findings may not be generalizable to infants of other ethnicities, preterm populations, or to longer-term growth and metabolic outcomes. More evidence from population trials is still needed. Second, recruitment was performed by several pediatricians, which could introduce some variation in the measurements performed. To ensure comparability of the anthropometric data obtained among sites, all pediatricians were trained to follow the same standardized procedures for anthropometrics, while intra- and inter-observer reliability was also periodically assessed. Finally, minor differences existed between the study formulas beyond protein hydrolysis, and the study design does not permit us to elaborate on the different effects of each of these minor differences separately.

## 5. Conclusions

In conclusion, this randomized controlled trial demonstrates that an infant formula containing partially hydrolyzed whey and intact protein supports adequate and sustained growth and is well-tolerated in healthy full-term infants during the first six months of life. Importantly, growth trajectories associated with the pHF were comparable to those observed in breastfed infants, and they can support linear growth during early infancy without promoting excessive weight gain.

## Figures and Tables

**Table 1:** Main ingredients and nutrient composition of the two infant formulas in this study (per 100 g powder).

	Intervention Formula	Control Formula
Energy, kJ	2123	2137
Protein *, g	10.7	11.1
Intact whey protein, g	2.2	6.7
Hydrolyzed whey protein, g	4.2	/
Intact casein, g	4.3	4.4
Whey: casein	6:4	6:4
Fat, g	26.6	27.3
Linoleic acid, g	3.9	3.9
α-Linolenic acid, mg	420	490
DHA, mg	106	105
ARA, mg	127	124
1,3-Dioleoyl-2-palmitoyl-glycerol (OPO), g	6	6.5
Carbohydrate, g	55.5	54.4
lactose	≥90%	≥90%
Fructo-oligosaccharides (FOSs), mg	690	850
Galacto-oligosaccharides (GOSs), mg	1033	850
Vitamin A, μg RE	369	369
Vitamin D, μg	12.5	14.2
Vitamin E, mg α-TE	7.1	7.0
Vitamin K_1_, μg	34	26
Vitamin B_1_, μg	355	510
Vitamin B_2_, μg	661	1158
Vitamin B_6_, μg	210	323
Vitamin B_12_, μg	1.7	0.7
Niacin, μg	3310	3280
Folic acid, μg	135	135
Pantothenic acid, μg	3340	3380
Vitamin C, mg	55	105
Calcium, mg	268	273
Phosphorus, mg	190	203
Iron, mg	2.8	3.0
Casein phosphopeptides (CPPs), mg	30	/

Intervention formula: Partially hydrolyzed whey protein infant formula; Control formula: standard cow’s milk protein infant formula. *: Protein sources and percentages of total protein in the intervention formula: 39.57% Lacprodan^®^ IF-3070; 3.64% Lacprodan^®^ ALPHA-10, 2.60% Lacprodan^®^ MFGM-10 and 1.45% Lacprodan^®^ DI-2021.

**Table 2:** Demographic, perinatal, and baseline characteristics of study infants.

Characteristics	pHF (*n* = 78)	SF (*n* = 70)	Before PSM		After PSM	
			BF (*n* = 103)	*p*	BF (*n* = 70)	*p*
Infant characteristics						
Age at baseline (days)	7.0 (4.0, 17.0) ^a^	7.0 (3.0, 13.0) ^a^	3.0 (2.0, 7.0) ^b^	**<0.001**	4.0 (2.0, 10.8)	0.091
Gender (male), *n* (%)	42 (53.8%)	43 (61.4%)	53 (51.5%)	0.420	39 (55.7%)	0.631
Baseline weight, g	3295 ± 451	3353 ± 450	3371 ± 433	0.505	3321 ± 466	0.741
Baseline length, cm	50.0 (49.0, 50.0)	50.0 (49.0, 50.0)	50.0 (49.0, 50.0)	0.626	50.0 (48.0, 50.0)	0.370
Baseline head circumference, cm	34.0 (33.0, 34.0)	34.0 (33.0, 34.00)	34.0 (33.0, 34.3)	0.646	34.0 (33.0, 34.0)	0.679
Birth weight, g	3151 ± 364 ^a^	3308 ± 426 ^b^	3311 ± 376 ^b^	**0.012**	3247 ± 399	0.053
Birth length, cm	50.0 (49.0, 50.0)	50.0 (49.0, 50.0)	50.0 (48.5, 50.0)	0.451	49.0 (48.0, 50.0)	0.218
Birth head circumference, cm	34.0 (33.0, 34.0) ^a^	34.0 (33.0, 34.0) ^a,b^	34.0 (33.0, 34.5) ^b^	**0.036**	34.0 (33.0, 34.4)	0.140
Gestational age (weeks)	38.7 ± 1.2 ^a^	39.1 ± 1.1 ^a,b^	39.3 ± 1.0 ^b^	**0.002**	39.0 ± 1.0	0.100
Caesarean delivery, *n* (%)	38 (48.7%) ^a^	36 (51.4%) ^a^	30 (29.4%) ^b^	**0.005**	28 (40.0%)	0.365
Maternal characteristics						
Age at baseline (years)	31.7 ± 5.7	30.1 ± 5.3	30.6 ± 4.2	0.156	30.6 ± 4.3	0.179
Area, *n* (%)				0.093		0.629
Changsha	40 (51.3%)	36 (51.4%)	71 (68.9%)		44 (62.9%)	
Chenzhou *	7 (9.0%)	4 (5.7%)	8 (7.8%)		6 (8.6%)	
Hengyang	10 (12.8%)	11 (15.7%)	11 (10.7%)		9 (12.9%)	
Zhuzhou	3 (3.8%)	2 (2.9%)	4 (3.9%)		3 (4.3%)	
Xingtai	18 (23.1%)	17 (24.3%)	9 (8.7%)		8 (11.4%)	
Ethnicity (minority), *n* (%)	1 (1.3%)	5 (7.1%)	6 (5.8%)	0.191	3 (4.3%)	0.193
Education level, *n* (%)				**<0.001**		**0.005**
<high school	23 (29.5%) ^a^	22 (31.4%) ^a^	10 (9.8%) ^b^		9 (12.9%)	
high school	35 (44.9%) ^d^	33 (47.1%) ^d^	40 (39.2%)		28 (40.0%) ^e^	
≥university degree	20 (25.6%)	15 (21.4%)	52 (51.0%)		33 (47.1%)	
Monthly per capita household income, CNY, *n* (%)				**0.001**		**0.046**
≤3000	44 (56.4%) ^a^	41 (58.6%) ^a^	32 (31.4%) ^b^		26 (37.7%)	
3001–6000	22 (28.2%)	23 (32.9%)	47 (46.1%)		27 (39.1%)	
>6000	12 (15.4%)	6 (8.6%)	23 (22.5%)		16 (23.2%)	
Pre-pregnancy smoking, *n* (%)	2 (2.6%)	2 (2.9%)	4 (3.9%)	0.904	4 (5.7%)	0.656
Secondhand smoke exposure during early pregnancy, *n* (%)	7 (9.2%)	7 (10.3%)	10 (10.1%)	0.972	6 (9.1%)	0.966
Pre-pregnancy BMI, kg/m^2^	21.16 (19.59, 23.99)	22.29 (19.40, 26.12)	21.94 (19.98, 23.68)	0.532	22.05 (20.03, 23.70)	0.542
Weight gain during pregnancy, kg	12.2 (10.0, 16.0)	13.8 (10.1, 17.0)	13.0 (10.0, 16.0)	0.713	13.0 (10.3, 17.0)	0.699
Prenatal BMI, kg/m^2^	26.92 (24.44, 29.51)	27.88 (24.42, 31.15)	26.75 (25.18, 29.36)	0.667	26.85 (25.17, 30.00)	0.647
Primipara, *n* (%)	25 (32.1%)	23 (32.9%)	39 (37.9%)	0.670	27 (38.6%)	0.669

Abbreviations: pHF: Partially hydrolyzed whey protein infant formula. SF: Standard cow’s milk protein infant formula. BF: Breastfeeding. BMI: body mass index (calculated as weight in kilograms divided by height in meters squared), kg/m^2^. PSM: Propensity score matching. Matching variables include: infant age at baseline, birth weight, gestational age and delivery mode. Data were described by the mean ± SD or median (IQR) for continuous variables and frequency (percentages %) for categorical variables. Continuous variables were compared using one-way ANOVA or the Kruskal-Wallis test as appropriate, followed by Dunn’s post-hoc test; categorical variables were compared using the chi-square or Fisher’s exact test. Groups that do not share a common superscript letter differ (^a,b^ indicate comparisons between groups before matching; ^d,e^ indicate comparisons between groups after matching), *p* < 0.05. *: Chenzhou includes two centers.

**Table 3:** Weight gain of study infants from baseline to the 3- and 6- month follow-up.

Group	Weight Gain (g/d)	Adjusted Mean Difference Between Groups (pHF vs. SF)		
	LS Mean (SE)	Estimate	95% CI	*p*
Baseline—3 months follow-up				
BF	39.9 (1.97)	-	-	-
pHF	33.9 (1.81)	1.05	(−2.16, 4.27)	0.518
SF	32.6 (1.92)			
Baseline—6 months follow-up				
BF	26.4 (1.27)	-	-	-
pHF	26.0 (1.19)	0.64	(−1.55, 2.83)	0.562
SF	25.3 (1.27)			

ANCOVA analysis was also conducted. Adjusted for region, infant’s gender, birth weight, age at enrollment, delivery mode, gestational age at delivery, maternal education level and family economic status. CI: Confidence interval; LS mean: Least squares mean; SE: Standard error; BF: Breastfeeding; pHF: Partially hydrolyzed whey protein infant formula; SF: Standard cow’s milk protein infant formula.

**Table 4:** Associations between different feeding groups and WHO growth standard z-scores over time.

Outcomes	Crude Model		Adjusted Model ^a^	
	*β* Coefficient (95% CI)	*p*	*β* Coefficient (95% CI)	*p*
**WAZ**				
group				
pHF	0 (reference)	-	0 (reference)	-
BF	0.33 (0.04, 0.63)	**0.028**	0.26 (0.01, 0.50)	**0.039**
SF	0.24 (−0.05, 0.54)	0.107	0.16 (−0.07, 0.40)	0.174
time	0.24 (0.18, 0.29)	**<0.001**	0.24 (0.18, 0.29)	**<0.001**
group × time				
pHF	0 (reference)	-	0 (reference)	-
BF	0.01 (−0.07, 0.09)	0.750	0.01 (−0.07, 0.09)	0.773
SF	−0.08 (−0.15, 0.002)	0.058	−0.08 (−0.15, 0.001)	0.053
**LAZ**				
group				
pHF	0 (reference)	-	0 (reference)	-
BF	0.23 (−0.17, 0.63)	0.257	0.09 (−0.28, 0.47)	0.627
SF	0.28 (−0.12, 0.68)	0.172	0.15 (−0.21, 0.51)	0.419
time	0.29 (0.21, 0.37)	**<0.001**	0.29 (0.21, 0.36)	**<0.001**
group × time				
pHF	0 (reference)	-	0 (reference)	-
BF	−0.04 (−0.16, 0.07)	0.448	−0.04 (−0.15, 0.07)	0.449
SF	−0.18 (−0.29, −0.07)	**0.002**	−0.17 (−0.28, −0.06)	**0.003**
**WLZ**				
group				
pHF	0 (reference)	-	0 (reference)	-
BF	0.21 (−0.18, 0.59)	0.291	0.25 (−0.12, 0.62)	0.183
SF	0.10 (−0.28, 0.48)	0.605	0.11 (−0.24, 0.47)	0.530
time	0.13 (0.04, 0.21)	**0.003**	0.13 (0.05, 0.22)	**0.002**
group × time				
pHF	0 (reference)	-	0 (reference)	-
BF	0.05 (−0.07, 0.17)	0.417	0.05 (−0.07, 0.17)	0.412
SF	0.02 (−0.10, 0.14)	0.731	0.01 (−0.11, 0.14)	0.828
**HCZ**				
group				
pHF	0 (reference)	-	0 (reference)	-
BF	0.31 (−0.07, 0.68)	0.108	0.22 (−0.15, 0.59)	0.242
SF	0.08 (−0.30, 0.45)	0.692	−0.01 (−0.37, 0.34)	0.939
time	0.21 (0.14, 0.29)	**<0.001**	0.21 (0.14, 0.28)	**<0.001**
group × time				
pHF	0 (reference)	-	0 (reference)	-
BF	0.08 (−0.03, 0.18)	0.158	0.08 (−0.02, 0.19)	0.132
SF	−0.04 (−0.15, 0.06)	0.445	−0.04 (−0.14, 0.07)	0.494

Linear mixed effects model was performed with group and time as fixed factors, infants as the random effect and group × time as the interaction term. ^a^. Adjusted models were controlled for region, enrolled age, gender, birth weight, gestation age, maternal education level, family economic status. WHO, World Health Organization; WAZ, weight-for-age z score; LAZ, length-for-age z score; WLZ, weight-for-length z score; HCZ, head circumference-for-age z score. pHF: Partially hydrolyzed whey protein infant formula; SF: Standard cow’s milk protein infant formula. BF: Breastfeeding.

**Table 5:** Overall adjusted mean differences (95% CI) in WHO growth standard z-scores among different feeding groups (Tukey-adjusted comparisons) ^a^.

Outcomes	pHF vs. BF		pHF vs. SF		BF vs. SF	
	Difference (95% CI)	*p*	Difference (95% CI)	*p*	Difference (95% CI)	*p*
WAZ	−0.21 (−0.45 to 0.02)	0.080	0.12 (−0.10 to 0.34)	0.396	0.34 (0.10 to 0.58)	**0.003**
LAZ	0.05 (−0.33 to 0.43)	0.957	0.26 (−0.10 to 0.62)	0.202	0.22 (−0.18 to 0.61)	0.395
WLZ	−0.29 (−0.63 to 0.05)	0.109	−0.05 (−0.37 to 0.27)	0.916	0.24 (−0.11 to 0.58)	0.248
HCZ	−0.33 (−0.72 to 0.06)	0.115	0.16 (−0.21 to 0.52)	0.579	0.49 (0.09 to 0.89)	**0.013**

Linear mixed-effects model-based mean and 95% confidence interval (CI). Pairwise comparisons were Tukey-corrected for multiple comparisons. ^a^. Adjusted models were controlled for region, enrolled age, sex, birth weight, delivery method, delivery gestation age, maternal education level, family economic status. WHO, World Health Organization; WAZ, weight-for-age z score; LAZ, length-for-age z score; WLZ, weight-for-length z score; HCZ, head circumference-for-age z score. pHF: partially hydrolyzed whey protein infant formula; SF: standard cow’s milk protein infant formula. BF: breastfeeding.

**Table 6:** Feeding behaviors during the study period in each group.

	Visits	pHF	SF	BF	*p*
Average number of feedings per day	1 month	8.0 (7.33, 9.67)	8.0 (7.17, 9.67)	9.33 (7.67, 10.33)	0.213
	2 months	8.0 (6.33, 8.67)	7.67 (6.33, 8.75)	8.33 (7.33, 9.50)	0.269
	3 months	6.83 (6.00, 7.33)	6.66 (5.67, 7.33)	7.67 (6.67, 9.00)	0.365
	6 months	5.83 (5.0, 6.0)	5.5 (5.0, 6.33)	6.33 (5.33, 7.67)	0.354
Average amount of study formula per day (mL/day) *	1 month	797 ± 165	780 ± 190	-	0.567
	2 months	879 ± 161	865 ± 231	-	0.674
	3 months	881 ± 206	928 ± 224	-	0.217
	6 months	946 ± 193	933 ± 229	-	0.743
Average energy intake from study formula (kJ/day)	1 month	2183.3 ± 452.8	2148.9 ± 523.3	-	0.686
	2 months	2408.3 ± 440.3	2383.5 ± 636.6	-	0.797
	3 months	2412.0 ± 563.5	2556.9 ± 617.7	-	0.165
	6 months	2592.1 ± 527.5	2572.4 ± 630.1	-	0.860
Adding complementary food and proportion, *n* (%)	3 months	1 (1.3%)	0 (0%)	1 (1.4%)	0.999
	6 months	37 (46.1%)	33 (46.4%)	27 (38.6%)	0.480

* Average daily consumed volume per visit was calculated if at least three diary days were filled out completely, including volume of water and number of scoops used and the volume of the leftover reported for each day. pHF: partially hydrolyzed whey protein infant formula; SF: standard cow’s milk protein infant formula; BF: breastfeeding.

**Table 7:** Average number of adverse events of each type in each group during the study period.

Group	pHF (*n* = 78)	SF (*n* = 70)	BF (*n* = 70)	*p*
Gastrointestinal disorders	0.73 ± 1.11 ^a,b^	0.81 ± 1.15 ^b^	0.39 ± 0.82 ^a^	**0.029**
Respiratory, thoracic and mediastinal disorders	0.63 ± 1.20	0.69 ± 1.23	0.40 ± 0.88	0.263
Allergic disorders	0.12 ± 0.36	0.06 ± 0.23	0.03 ± 0.17	0.175
General disorders	0.19 ± 0.51	0.20 ± 0.47	0.26 ± 0.56	0.730

Event counts were compared using Kruskal–Wallis tests, followed by Dunn’s post hoc test. Groups that do not share a common superscript letter differ, *p* < 0.05. pHF: partially hydrolyzed whey protein infant formula; SF: standard cow’s milk protein infant formula; BF: breastfeeding. ^a,b^: Groups that do not share a common superscript letter differ.

## Data Availability

The data that support the findings of this study are available from the corresponding author upon reasonable request. Due to ethical and privacy considerations involving infant participants, the dataset is not publicly available.

## Supplementary Materials

The following supporting information can be downloaded at https://www.mdpi.com/article/10.3390/nu18050770/s1, Table S1: Weight Gain of Study Infants from Baseline to the 3- and 6- Month Follow-up before propensity score matching; Table S2: Anthropometric measurements at each follow-up time point in each study group; Figure S1: Mean changes in World Health Organization growth standard z-scores weight-for-age (A), length-for-age (B), weight-for-length (C), and head circumference-for-age (D) over time by different feeding groups.
