# Supplementary material for: An Infant Formula with Partially Hydrolyzed Whey and Intact Protein Demonstrates Adequate Growth and Safety: A 6-Month Randomized, Triple-Blind, Controlled Trial
Source: Nutrients. 2026 Feb 26;18(5):770. doi: 10.3390/nu18050770 (PMC12986564; doi:10.3390/nu18050770)
Supplement: Supplementary file 1 [file nutrients-18-00770-s001.zip › nutrients-4121924-supplementary.pdf]

Table S1. Weight Gain of Study Infants from Baseline to the 3- and 6- Month Follow-up before propensity score matching.

**Group**	**Weight gain (g/d)**	**Adjusted mean difference between groups (pHF vs SF)**		
	**LS mean (SE)**	**Estimate**	**95% CI**	***P***
	Baseline - 3 months follow-up			
BF	39.8 (2.94)	-	-	-
pHF	34.3 (2.87)	1.29	(-1.91, 4.49)	0.427
SF	33.0 (2.81)			
	Baseline - 6 months follow-up			
BF	24.2 (2.10)	-	-	-
pHF	23.9 (3.02)	0.86	(-1.38, 3.10)	0.449
SF	23.0 (3.14)			

ANCOVA analysis was also conducted. Adjusted for region, infant's gender, baseline weight, age at enrollment, delivery mode, gestational age at delivery, maternal education level and family economic status.

CI: Confidence interval; LS mean: Least squares mean; SE: Standard error; BF: breastfeeding; pHF: Partially hydrolyzed whey protein infant formula; SF: Standard cow's milk protein infant formula.

Table S2. Anthropometric measurements at each follow-up time point in each study group

	**pHF (n = 78)**	**SF (n = 70)**	**BF (n = 70)**	***P***
**Weight (g)**				
Baseline	3295 ± 451	3353 ± 450	3321 ± 466	0.609
1 month	4547 ± 511	4728 ± 684	4599 ± 583	0.132
2 months	5635 ± 659	5707 ± 779	5926 ± 740	0.094
3 months	6589 ± 781	6561 ± 1040	6897 ± 830	0.052
6 months	8268 ± 1057	8263 ± 1155	8231 ± 1099	0.978
**Length (cm)**				
Baseline	49.7 ± 1.7	49.9 ± 1.7	49.5 ± 1.8	0.370
1 month	54.1 ± 2.1	54.8 ± 3.2	54.2 ± 2.5	0.265
2 months	58.5 ± 3.2	58.0 ± 4.0	58.4 ± 2.7	0.697
3 months	61.8 ± 3.4	61.4 ± 3.2	61.9 ± 2.9	0.835
6 months	68.0 ± 3.9	67.3 ± 3.3	67.4 ± 3.2	0.505
**Head circumference (cm)**				
Baseline	33.8 ± 1.4	33.7 ± 1.1	33.9 ± 1.3	0.679
1 month	36.6 ± 1.7	36.9 ± 1.9	36.9 ± 1.3	0.592
2 months	38.6 ± 1.7	38.5 ± 1.9	38.8 ± 1.6	0.397
3 months	39.9 ± 1.6	40.1 ± 1.9	40.4 ± 1.5	0.307
6 months	42.7 ± 1.6	42.7 ± 1.7	43.3 ± 1.9	0.249

pHF: Partially hydrolyzed whey protein infant formula; SF: Standard cow's milk protein infant formula; BF: breastfeeding.

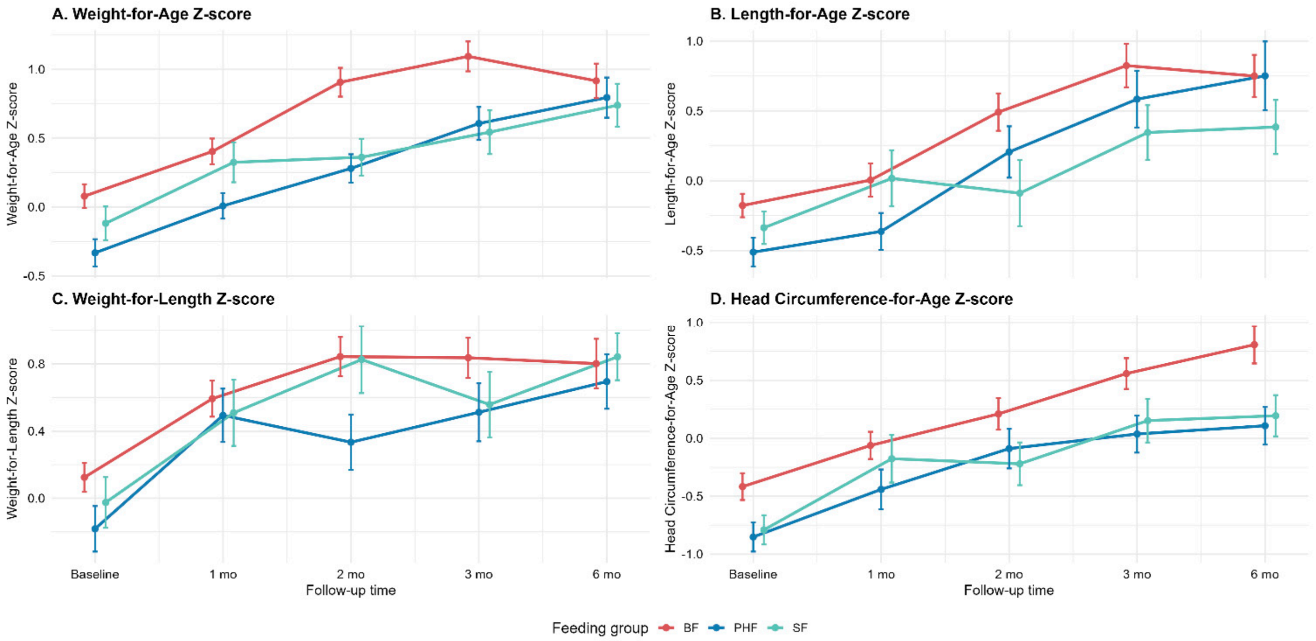

Figure S1. Mean Changes in World Health Organization Growth Standard z-scores Weight-for-age (A), Length-for-age (B), Weight-for-length (C), Head circumference-for-age (D) Over Time by Different Feeding Groups
